# Comparison of Soybean Transformation Efficiency and Plant Factors Affecting Transformation during the *Agrobacterium* Infection Process

**DOI:** 10.3390/ijms160818522

**Published:** 2015-08-07

**Authors:** Yuying Jia, Xingdong Yao, Mingzhe Zhao, Qiang Zhao, Yanli Du, Cuimei Yu, Futi Xie

**Affiliations:** Soybean Research Institute, Shenyang Agricultural University, Shenyang 110866, China; E-Mails: jiayuyinggood@163.com (Y.J.); yaoxingdong@gmail.com (X.Y.); soyshen@163.com (M.Z.); zqiang1987@126.com (Q.Z.); dyl0305@sina.cn (Y.D.)

**Keywords:** soybean, transformation, cotyledonary node, *Agrobacterium* infection, plant factors

## Abstract

The susceptibility of soybean genotype to *Agrobacterium* infection is a key factor for the high level of genetic transformation efficiency. The objective of this study is to evaluate the plant factors related to transformation in cotyledonary nodes during the *Agrobacterium* infection process. This study selected three genotypes (Williams 82, Shennong 9 and Bert) with high transformation efficiency, which presented better susceptibility to *Agrobacterium* infection, and three low transformation efficiency genotypes (General, Liaodou 16 and Kottman), which showed a relatively weak susceptibility. Gibberellin (GA) levels and soybean *GA20ox2* and *CYP707A2* transcripts of high-efficiency genotypes increased and were higher than those of low-efficiency genotypes; however, the opposite performance was shown in abscisic acid (ABA). Higher zeatin riboside (ZR) content and DNA quantity, and relatively higher expression of soybean *IPT5*, *CYCD3* and *CYCA3* were obtained in high-efficiency genotypes. High-efficiency genotypes had low methyl jasmonate (MeJA) content, polyphenol oxidase (PPO) and peroxidase (POD) activity, and relatively lower expression of soybean *OPR3*, *PPO1* and *PRX71*. GA and ZR were positive plant factors for *Agrobacterium*-mediated soybean transformation by facilitating germination and growth, and increasing the number of cells in DNA synthesis cycle, respectively; MeJA, PPO, POD and ABA were negative plant factors by inducing defence reactions and repressing germination and growth, respectively.

## 1. Introduction

Over the past decade, genetically modified soybean has continued to be the predominant commercialized biotech crop, reaching 75.4 million hectares (almost 50% of the total worldwide biotech crop area) in 2011 [1]. Transgenic technology provides an attractive alternative to conventional soybean breeding programs, especially in introducing valuable agronomic traits such as herbicide and pest resistance [2,3]. However, high-efficiency transgenic soybean methodologies still need to be developed for many elite soybean lines which are insusceptible to *Agrobacterium* infection [4,5].

Most plant transformation systems are lengthy processes comprised of multiple complicated steps. *Agrobacterium*-mediated transformation is proved to be a superior soybean transformation method over other transformation systems because it offers significant advantages including easier manipulation, lower transgene copy number and greater transgene stability, which frequently result in a higher transformation success rate [6]. To generate a stable *Agrobacterium*-mediated transgenic soybean line, foreign genes (contained within T-DNA) are delivered from *Agrobacterium* into plant host cells and eventually integrated into the host genome. This infection process is a very critical early step of the whole transformation process beginning with recognition of plant signals by *Agrobacterium*, followed by *Agrobacterium* attachment to the wounded plant tissue and the survival of imported T-DNA from the host defense system [7]. Therefore, successful *Agrobacterium* infection is necessary for ensuring a high level of soybean transformation efficiency, which depends on both plant genotypes [5,8] and *Agrobacterium* strains [9,10]. Compared with systematically studied *Agrobacterium* events during infection [11,12,13], our understanding on the host plant events has mostly focused on T-DNA and biochemical compounds. Multiple studies have identified a set of plant proteins and genes which involve T-DNA import, transport and integration into the plant genome [14,15,16,17,18]. On the other hand, a number of plant cell secreted compounds have been shown to affect infection through inducing or inhibiting the expression of *Agrobacterium* virulence genes [19,20,21]. Although great success has been achieved in characterization of plant factors affecting the *Agrobacterium* infection process, more efforts are required to investigate details on the delicate plant cellular response during *Agrobacterium* infection.

Since the successful transformation of the cultivated soybean by *Agrobacterium*-mediated method in 1988 [22], a lot of researchers discussed the conditions affecting soybean transformation to optimize the soybean transformation system [8,23,24,25]. Improved transformation systems enhanced the soybean transformation efficiency, but the degree of improvement was limited. Moreover, some studies showed that in soybean genotypes existed a variation in susceptibility to *Agrobacterium* [4], and many researchers mainly focused on screening soybean cultivars of high transformation efficiency. Soybean cultivar Williams 82 was commonly used in soybean transformation, usually as a control. Jack, Peking and Bert were also suitable cultivars in different transformation culture conditions [10,23,26,27]. Researchers also identified some Chinese cultivars with a stable transgenic efficiency [5,25].

Studies optimizing *Agrobacterium*-mediated transformation, the addition of phenolic compounds [28], antioxidants [29,30] and phytohormones [31,32] partly enhanced the transformation. Phenolic compounds, like acetosyringone (AS), have been found to be essential for induction of the virulence gene [20]. These antioxidant reagents (l-cysteine, thiol compounds and dithiothreitol) appeared to improve T-DNA delivery by inhibiting the activity of plant pathogen-response and wound-response enzymes, such as polyphenol oxidase (PPO) and peroxidase (POD) [33], but did not fundamentally repress plant defense reaction to *Agrobacterium* infection. In plant tissue culture, adding phytohormones usually caused changes in endogenous hormones. Endogenous hormones played essential roles in regulating plant growth, development, and stress responses. Gibberellin (GA) is known to induce the germination process and promote degradation of storage material in seeds [34], providing the material and energy basis for the explants. Abscisic acid (ABA) not only acted as an antagonist to GA [35,36], but also had an involvement in responses to flooding, pathogen attack and wounding [37]. Methyl jasmonate (MeJA) involved in plant development and the regulation in the expression of plant defense genes in response to various stresses such as wounding, drought, and pathogens [38]. Bacterial infection and wounding are necessary in *Agrobacterium*-mediated transformation systems, MeJA may participates in this process. Other phytohormones such as cytokinin were required to induce cell division and growth in plant tissue cultures. Villemont *et al.* [39] investigated that *Petunia* mesophyll cycling cells with no phytohormone treatments could not be transformed. Thus, efficient *Agrobacterium* transformation might occur at a particular stage of the plant cell cycle. The previous researches implied that germination and growth, cell division and defense response status of explant tissues might be a crucial effect on the transformation.

Although a lot of progress has been made in soybean transformation, transgenic efficiency still requires improvement. Most screened cultivars had a shortcoming on agronomic traits. Genotype-dependency of the soybean transformation significantly limited its application of elite and commercially valuable cultivars. It took a long time for breeding a commercial transgenic soybean after getting a transgenic plant in the laboratory. If researchers can get high transformation efficiency at any time from elite and commercially valuable cultivars, the transgenic breeding efficiency would be improved greatly. As soybean genotypes display a variation in susceptibility to *Agrobacterium* [4], which plant factors are involved? To the best of our knowledge, there has been no report concerning the identification and analysis of plant factors from soybean. The objective of this study is to evaluate the plant factors related to transformation in cotyledonary nodes during the *Agrobacterium* infection process as based on the screened soybean cultivars with contrasting transformation efficiencies. Our data suggest that GA and ZR play a positive role on *Agrobacterium*-mediated soybean transformation; however, ABA, MeJA, PPO and POD have a negative effect. Furthermore, these factors participated in germination and growth, cell division and defense response in cotyledonary nodes.

## 2. Results

### 2.1. Transformation Efficiencies of Various Soybean Cultivars

**Figure 1:**
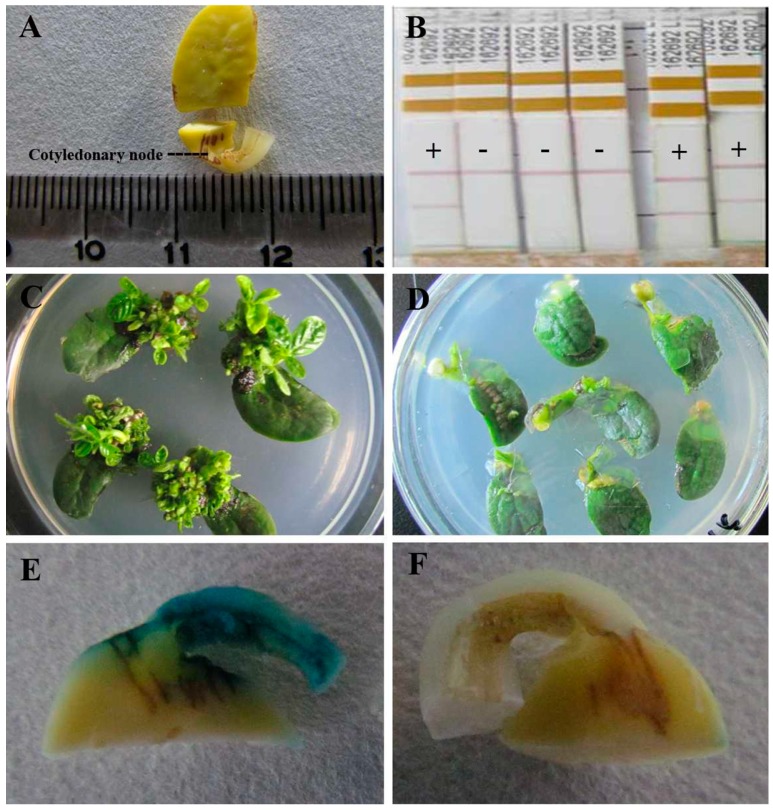
Cotyledonary node for determination, LibertyLink strip analysis of T_0_ plants, phenotype of explants after shoot induction, and GUS staining after co-cultivation for five days. (**A**) Sampling standard of cotyledonary node, numbers on the ruler represent centimeters; (**B**) Transgenic soybean plants were tested using LibertyLink strips, +: positive T_0_ plants, −: negative T_0_ plants; (**C**) Explants with phosphinothricin-resistant multiple buds; (**D**) Explants with no buds; (**E**) Cotyledonary node after GUS staining dyeing; and (**F**) Cotyledonary node with no GUS staining.

There was a significant transformation difference among ten given cultivars (Table 1). After selection with 5 mg·L^−1^ phosphinothricin on shoot induction medium for four weeks, resistant explants to phosphinothricin produced multiple observable buds (Figure 1C), while non-resistant explants had no buds (Figure 1D). Williams 82 grew well and had 80.69% resistant shoot induction rate, followed by Shennong 9 and Bert, while General, Liaodou 16 and Kottman showed significantly inferior resistant shoot induction rate. Transformation efficiencies of T_0_ plants of Williams 82, Shennong 9 and Bert were 6.71%, 5.32% and 5.13%, respectively, which were superior to other cultivars. In addition, transformation efficiencies of Dennison, Kottman, General and Liaodou 16 were very poor, below 1%, and Liaodou 16 even did not produce a positive plant. Based on the transformation efficiency, we screened three high-efficiency genotypes including Williams 82, Shennong 9 and Bert and three low-efficiency genotypes such as General, Liaodou 16 and Kottman. These genotypes had contrasting transformation efficiencies, and thus were used in the subsequent experiments.

**Table 1:** Evaluation of 10 soybean cultivars for *Agrobacterium*-mediated transformation.

Cultivar	No. of Explants Infected	No. of Explants with above Three Shoots	Resistant Shoot Induction Rate (%)	No. of Positive T_0_ Plants	Transformation Efficiency of T_0_ Plants (%)
Liaodou16	348	11	3.2	0	0
General	481	46	9.6	1	0.21
Kottman	432	44	10.2	3	0.69
Dennison	348	67	19.3	3	0.86
Shennong 12	504	185	36.7	8	1.59
Liaodou 14	384	244	63.5	7	1.82
Liaodou 10	576	382	66.3	13	2.26
Bert	624	477	76.4	32	5.13
Shennong 9	564	436	77.3	30	5.32
Williams 82	492	397	80.7	33	6.71

Resistant shoot induction rate (%) = (No. of explants with at least three shoots/No. of explants infected) × 100; Transformation efficiency (%) = (No. of positive T_0_ plants/No. of explants infected) × 100.

### 2.2. Susceptibility of Different Soybean Genotypes to Agrobacterium Infection

**Figure 2:**
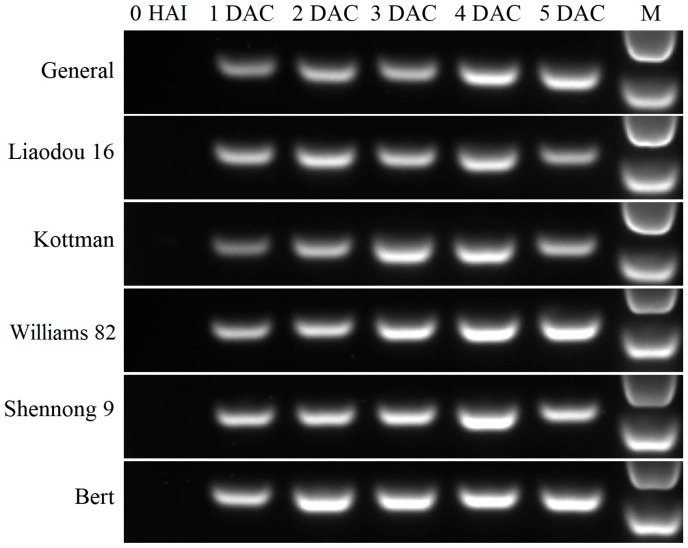
PCR analysis of *BAR* gene accumulation from different genotypes during co-cultivation period. Abbreviations represent different phases: 0 HAI (0 h after infection), 1 DAC (one day after co-cultivation), 2 DAC (two days after co-cultivation), 3 DAC (three days after co-cultivation), 4 DAC (four days after co-cultivation), and 5 DAC (five days after co-cultivation). M: DL2000 marker.

To determine the susceptibility of screened soybean genotypes to *Agrobacteriumin* infection, this study detected transient β-glucuronidase (GUS) expression and *BAR* gene accumulation of cotyledonary nodes during co-cultivation period. Major GUS staining tissues were around cotyledonary nodes, which were easily accessible to the *Agrobacterium*. According to the stained (Figure 1E) and unstained cotyledonary node (Figure 1F), transient GUS expression rates were calculated and listed in Table 2. General, Kottman and Liaodou 16 exhibited less than 40% staining, while Williams 82, Shennong 9 and Bert showed above 70%, indicating that high-efficiency genotypes had a higher transient GUS expression rate. As shown in Figure 2, the *BAR* gene detected by PCR was the sum of that in *Agrobacterium* and cotyledonary node cells during co-cultivation period. *BAR* gene was accumulated progressively after *Agrobacterium* infection. It is worth noting that Williams 82, Shennong 9 and Bert accumulated more *BAR* gene than General, Liaodou 16 and Kottman, especially at 3 days after co-cultivation (DAC) and 4 DAC, suggesting that *Agrobacterium* was more acceptable by high-efficiency genotypes. These results indicate that high-efficiency genotypes are more susceptible to *Agrobacterium* infection.

**Table 2:** Transient GUS expression in cotyledonary nodes from different soybean genotypes.

Genotype	No. of Cotyledonary Nodes for GUS Staining	No. of GUS^+^ Cotyledonary Nodes	GUS^+^ Rate (%)
General	98	32	32.7
Liaodou 16	84	16	19.1
Kottman	100	30	30.0
Williams 82	108	86	85.7
Shennong 9	98	84	79.6
Bert	104	80	76.9

Transient GUS expression rate (%) = (No. of cotyledonary nodes with GUS staining/No. of cotyledonary nodes for GUS staining) ×100.

### 2.3. Germination and Growth Related Factors Affecting Transformation

**Figure 3:**
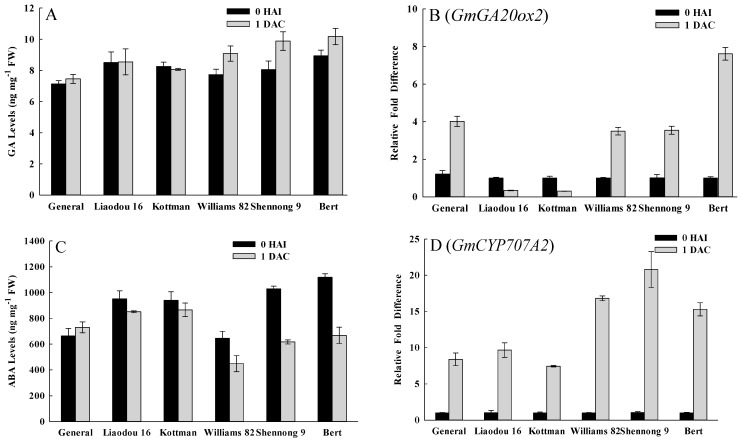
Comparisons of gibberellin (GA) and abscisic acid (ABA) levels, and the expression levels of genes ralated to GA and ABA metabolism between different genotypes. The values represent means ± SD based on three biological replications. (**A**) GA content in cotyledonary nodes after 0 h of infection and after 1 day of co-cultivation; (**B**) qRT-PCR analysis of GA synthetic gene *GmGA20ox2*; (**C**) ABA content; and (**D**) qRT-PCR analysis of ABA catabolic gene *GmCYP707A2*.

ABA acts as an antagonist to GA in regulating seed germination process [35,36]. Early increase in GA levels was thought to induce the germination process and promote degradation of storage material in seeds [34]. Cotyledonary node explants were also engaged in germinating and growing during co-cultivation period. In this study, prominent differences of GA and ABA levels were observed between high-efficiency genotypes and low-efficiency genotypes during the first day of co-cultivation (Table S1). GA levels increased in Williams 82, Shennong 9 and Bert, but changed slightly in General, Liaodou 16 and Kottman during the first day of co-cultivation (Figure 3A). In addition, GA content of Williams 82, Shennong 9 and Bert was markedly higher than that of General, Liaodou 16 and Kottman at 1 DAC. *GA20ox2* encodes an enzyme required for biosynthesis of GA [40]. Soybean *GA20ox2* transcripts increased in General, Williams 82, Shennong 9 and Bert at 1 DAC (Figure 3B), and this was roughly consistent with the changes of GA levels. In contrast with GA, ABA levels of all genotypes exhibited different degrees of decreased concentrations, where Williams 82, Shennong 9 and Bert had a lower ABA content than General, Liaodou 16 and Kottman (Figure 3C). In addition, the soybean homologue of the ABA catabolic gene *CYP707A2* [41,42] exhibited a 15-fold increase in expression in Williams 82, Shennong 9 and Bert, but below a 10-fold increase in General, Liaodou 16 and Kottman at 1 DAC (Figure 3D), manifesting a more rapid decrease of ABA levels in high-efficiency genotypes, which was in agreement with ABA variation. In conclusion, high-efficiency genotypes had higher GA levels and lower ABA levels at 1 DAC, which indicated that germination and growth occured more quickly in the early stage of co-cultivation.

### 2.4. Cell Division Related Factors Affecting Transformation

**Figure 4:**
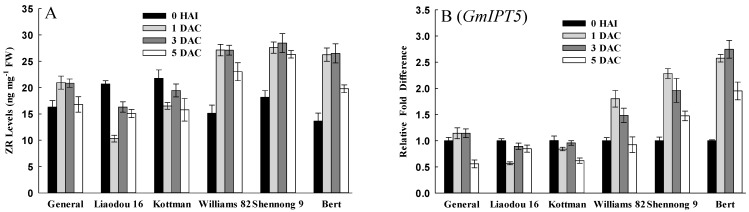
Comparison of zeatin riboside (ZR) levels and the expression levels of ZR synthetic gene between different genotypes. The values represent means ± SD based on three biological replications. (**A**) ZR content in cotyledonary nodes after 0 h of infection, and after one day, three days, and five days of co-cultivation; (**B**) qRT-PCR analysis of ZR synthetic gene *GmIPT5*.

Cytokinins are plant hormones that regulate plant cell division. To investigate the cell division differences of genotypes during co-cultivation period, ZR levels and the transcriptional levels of *IPT5* that encodes a rate limiting enzyme in cytokinin biosynthesis [43] were determined. As shown in Figure 4A, ZR concentration of Williams 82, Shennong 9 and Bert increased drastically after *Agrobacterium* infection, and remained at a relatively high level at 1 DAC and 3 DAC. While ZR levels in General, Liaodou 16 and Kottman increased slightly or declined. In addition, ZR content in Williams 82, Shennong 9 and Bert was significantly higher than that in General, Liaodou 16 and Kottman during the whole co-cultivation period. Expression patterns of soybean *IPT5* were in accordance with ZR accumulation in all genotypes. And the relative expression of *GmIPT5* in Williams 82, Shennong 9 and Bert were prominently higher than that in General, Liaodou 16 and Kottman, with a maximum at 1 DAC (Figure 4B). These results indicated that high-efficiency genotypes had higher cell division potential than low-efficiency genotypes.

**Figure 5:**
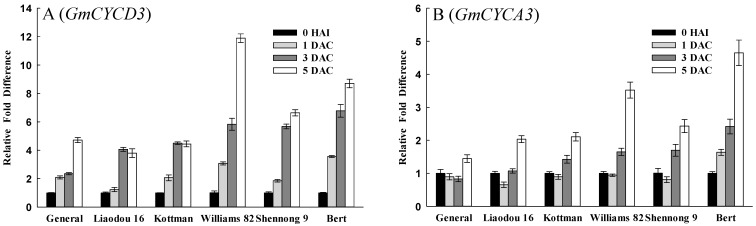
Comparison of the expression levels of cell cyclin-associated genes between different genotypes. The values represent means ± SD based on three biological replications. (**A**) qRT-PCR analysis of *GmCYCD3* in cotyledonary nodes after 0 h of infection, and after one day, three days, and five days of co-cultivation; (**B**) qRT-PCR analysis of *GmCYCA3*.

Cell division has a significant influence on *Agrobacterium*-mediated soybean transformation. To research the relationship between cell cycle phase and *Agrobacterium* transformation, this study investigated the expression of cell cycle-associated genes in different genotypes. *CYCD3* is the key for stimulating G1 and G1/S transition [44]. Soybean homologue of *CYCD3* was up-regulated during the co-cultivation period (Figure 5A). *CYCA3* is a S-phase specific expression gene in the next DNA synthesis cycle [45]. In this study, soybean *CYCA3* was up-regulated at 3 DAC and 5 DAC (Figure 5B). In addition, the expression of these two genes were increased more significantly in Williams 82, Shennong 9 and Bert at 3 DAC and 5 DAC, suggesting that high-efficiency genotypes had more cells entering into S-phase and being in S-phase as compared with low-efficiency genotypes in the later co-cultivation period.

**Figure 6:**
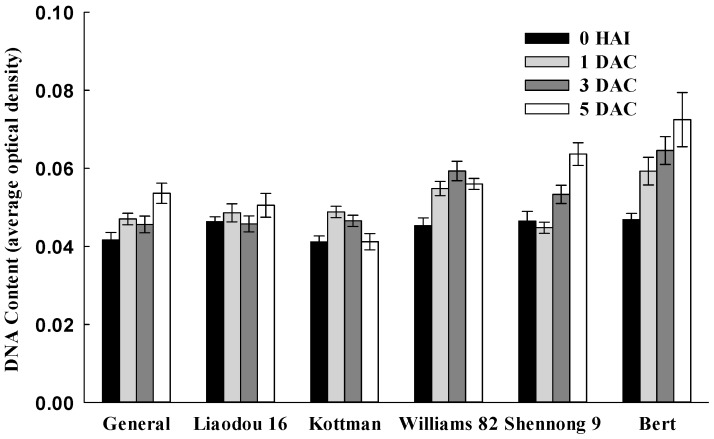
Comparison of nuclear DNA content in meristem cells of cotyledonary nodes between different genotypes during the co-cultivation period. The data represent the means ± SD of all cell nucleus in the cotyledonary node.

As the S-phase is part of the DNA synthesis cycle, this led us to test the nuclear DNA quantity in meristem cells of cotyledonary nodes. Fluorescence of DAPI-stained nuclei of different genotypes is presented in Figure S1. Williams 82, Shennong 9 and Bert displayed stronger DAPI fluorescence than General, Liaodou 16 and Kottman. Average optical density (AOD) is directly proportional to DNA content, further analysis are presented in Figure 6. DNA content of Williams 82, Shennong 9 and Bert exhibited up-trend during co-cultivation period, and significantly higher than those of General, Liaodou 16 and Kottman at 3 DAC and 5 DAC. The results indicated that soybean *CYCA3* expression pattern was same as the staining pattern with DAPI, which represented replicating DNA, suggesting a tangible link between S-phase and efficient *Agrobacterium* transformation.

### 2.5. Defense Response Related Factors Affecting Transformation

**Figure 7:**
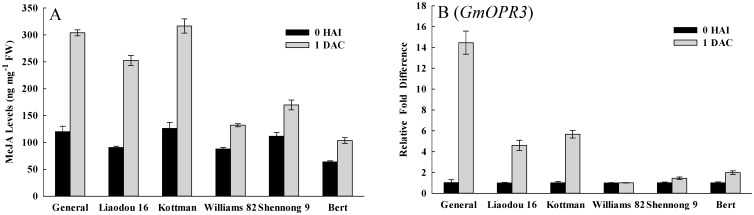
Comparison of methyl jasmonate (MeJA) levels and the expression levels of MeJA synthetic gene between different genotypes. The values represent means ± SD based on three biological replications. (**A**) MeJA content in cotyledonary nodes after 0 h of infection and after one day of co-cultivation; (**B**) qRT-PCR analysis of MeJA synthetic gene *GmOPR3*.

To study the responses of genotypes to wounding and *Agrobacterium* infection, MeJA levels and its synthetic gene *OPR3* [46] were investigated. After wounding and *Agrobacterium* infection, the peak of MeJA level was observed at 1 DAC. Williams 82, Shennong 9 and Bert all increased more than 2.5-fold, but General, Liaodou 16 and Kottman only increased up to 1.5-fold (Table S1). In addition, MeJA content of Williams 82, Shennong 9 and Bert was significantly higher than those of General, Liaodou 16 and Kottman at 1 DAC (Figure 7A). A soybean homologue of *OPR3* was strongly up-regulated in General, Liaodou 16 and Kottman at 1 DAC, especially increased 14-fold in General. However, the increase was less in Williams 82, Shennong 9 and Bert (below two-fold) (Figure 7B). The results indicated that wounding and *Agrobacterium* infection caused a significant increase in MeJA levels and *GmOPR3* expression of low-efficiency genotypes.

**Figure 8:**
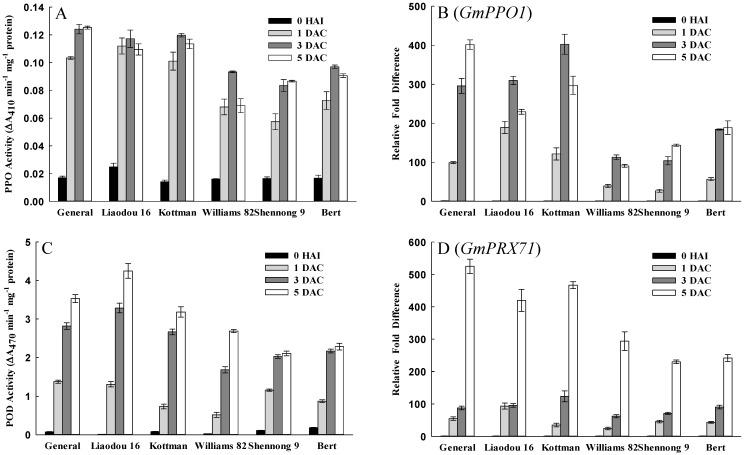
Comparison of polyphenol oxidase (PPO) and peroxidase (POD) activity, and the expression of encoding genes between different genotypes. The values represent means ± SD based on three biological replications. (**A**) PPO activity in cotyledonary nodes after 0 h of infection, and after one day, three days, and five days of co-cultivation; (**B**) qRT-PCR analysis of the gene *GmPPO1* that encodes PPO; (**C**) POD activity; and (**D**) qRT-PCR analysis of the gene *GmPRX71* that encodes POD.

PPO and POD are protective enzymes that respond to various stresses. In this study, PPO and POD activity and their encoding gene *PPO1* [47] and *PRX71* [48] were investigated. The results showed a striking increase of PPO and POD activity occurred during the co-cultivation period (Figure 8A,C). Moreover, PPO and POD activity in General, Liaodou 16 and Kottman was significantly higher than those in Williams 82, Shennong 9 and Bert at 3 DAC and 5 DAC. Expression of soybean homologue of *PPO1* and *PRX71* increased more significantly in General, Liaodou 16 and Kottman (Figure 8B,D), which followed a similar pattern with the variation of PPO and POD activity, respectively.

## 3. Discussion

There was a significant genotype dependency in *Agrobacterium*-mediated soybean transformation. This study screened three high-efficiency genotypes (Williams 82, Shennong 9 and Bert) and three low-efficiency genotypes (General, Liaodou 16 and Kottman) from T_0_ plants (Table 1). *Agrobacterium* infection plays a major role in the soybean transformation processes. Boyko *et al.* [49] demonstrated that the increase in transformation frequency was primarily due to the increase in transgene integration efficiency rather than in tissue regeneration efficiency. In this research, we employed transient GUS expression and *BAR* gene accumulation to evaluate six genotypes’ susceptibility to *Agrobacterium* infection. Soybean cultivar Williams 82 was commonly used in transformation research, which showed a relatively good performance in both transient GUS expression and *BAR* gene accumulation in this study, as well the genotypes Shennong 9 and Bert also performed well (Table 2 and Figure 2). Three low-efficiency genotypes presented poor transient GUS expression and *BAR* gene accumulation. Song *et al.* [5] also utilized transient GUS expression and resistant shoot regeneration rate to evaluate genotypes’ susceptibility to *Agrobacterium* infection. In our study, high-efficiency genotypes displayed higher resistant shoot regeneration rate (Table 1). These results strongly supported that high-efficiency genotypes possessed greater susceptibility to *Agrobacterium* infection. Increasing a genotypes’ susceptibility was an important prerequisite to improve the transgenetic efficiency of recalcitrant genotypes. Therefore, this study explored some plant factors which influence *Agrobacterium* infection during co-cultivation period.

In our study, GA levels and soybean *GA20ox2* and *CYP707A2* transcripts of high-efficiency genotypes increased and were higher than that of low-efficiency genotypes at 1 DAC (Figure 3A,B,D). However, the variation of ABA showed the opposite response (Figure 3C). This implied that storage material of high-efficiency genotypes are broken down more rapidly to provide the considerable energy and nutrients needed for the growth and differentiation of the cotyledonary node. Iglesias and Babiano [50] found that mature dry seeds of chickpea contained high levels of ABA, which decreased to a very low level after germination. But our results showed that ABA content of low-efficiency genotypes reduced very little at 1 DAC (Figure 3C), may be due to a stronger response to *Agrobacterium* infection. This is in accordance with the result that ABA had an involvement in the responses to pathogen attack and wounding [37].

In *Agrobacterium*-mediated transformation systems, it is necessary to wound the cotyledonary node to release phenolic compounds and provide access to the target cell [20]. On the one hand, wounding enhances endogenous cytokinins activity to stimulate cell division [51,52]; on the other hand, wounded tissues make jasmonates (JA) or MeJA, inhibiting cell division [53,54]. Compelling results were shown in this study, on the first day after wounding and *Agrobacterium* infection. ZR content drastically increased in high-efficiency genotypes, but did not in low-efficiency genotypes (Figure 4A). A concurrent increase in MeJA levels was observed in low-efficiency genotypes (Figure 7A). Taken together, these data indicated that different response of genotypes to wounding resulted in the variation of different endogenous hormones, an affect on cell division. Thus, cell division was activated in high-efficiency genotypes, but may be repressed in low-efficiency genotypes. Subsequently, the expression status of soybean cell cycle-associated genes *CYCD3* and *CYCA3* (Figure 5) suggested that the cells, which was in S-phase or would be in S-phase, were less in low tansformation efficiency genotypes. The increase of MeJA levels might be correlated with cell cycle, and produce a negative impact on G1/S transition and S-phase in low-efficiency genotypes. Actually, Patil *et al.* [55] also discovered that MeJA elicitation can inhibit cell cycle progression by impairing G1/S transition and decreasing S-phase cells in cultured *Taxus* cells.

Cytokinins can activate cell division [52], and many reports have shown a direct link between cytokinins and cell cycle control. For example, the peak of zeatin (Z) levels was detected at G1/S, G2/M and middle-late of S-phase in synchronized tobacco cell [56,57]. This study also showed that ZR content and expression of soybean *CYCD3* and *CYCA3* appeared to have a synchronous increase only in high-efficiency genotypes (Figure 4 and Figure 5), suggesting that cell cycle had a significant influence on soybean transformation. Chateau *et al.* [32] suggested that efficient *Agrobacterium* transformation might occur at a particular stage of the plant cell cycle. Villemont *et al.* [39] demonstrated that *Petunia* mesophyll cells could not be transformed when the cell cycle was blocked in late G1 phase, and cells with the highest transformation ability were those that had a very high ratio of S + G2 phase/M phase. This study also showed that higher expression of soybean *CYCD3* and *CYCA3*, and higher DNA content were obtained in high-efficiency genotypes at a later co-cultivation period (Figure 5 and Figure 6), which implied that high-efficiency genotypes had more cells in S-phase, in which cells were being DNA replication. These findings strongly supported the notion that successful *Agrobacterium*-mediated transformation required DNA replication in S-phase. We speculated that DNA replication might provide a good opportunity for the insertion of exogenous genes.

Explants regard *Agrobacterium* as invaders in the transformation process, and produce a defense response to battle *Agrobacterium* infection and expression of foreign genes. In addition, wounding also induces a defense reaction, including production of protective enzymes, induction of defence-related genes and so on. In this study, after wounding and *Agrobacterium* infection, MeJA content of cotyledonary nodes increased rapidly especially in low-efficiency genotypes (Figure 7A). MeJA triggered defense response to induce the production of defensive proteins, protective enzymes and phenolic acids that inhibited the infection of the pathogen [58]. *OPR3* can be induced by a variety of stimuli such as wounding and JA [59,60], and enhances resistance to necrotrophic fungus in *Arabidopsis* [61]. In this study, expression of soybean *OPR3* was also increased in low-efficiency genotypes (Figure 7B), which was consistent with the observation of MeJA. The current study with higher expression of soybean *OPR3* and MeJA concentration in low-efficiency genotypes, taken together with previous investigations, indicate that low-efficiency genotypes possess a higher defence ability in the *Agrobacterium* infection process. In addition, protective enzymes, PPO and POD are involved in reactions culminating in wound-induced tissue browning and participate in defense reactions against pathogenic invasion [62,63]. The data here showed that PPO and POD activity, and the expression of soybean *PPO1* and *PRX71*, all increased remarkably, and were significantly higher in low-efficiency genotypes (Figure 8), suggesting that there were intense defense reactions in low-efficiency genotypes. In phenol metabolism, PPO and POD could oxidate polyphenols into quinones (antimicrobial compounds), which were toxic for *Agrobacterium*, and typically resulted in extensive tissue browning and partial cell death to battle the infection process of *Agrobacterium*. These results strongly demonstrate that intense defense response of explant is a fatal weakness for *Agrobacterium*-mediated soybean transformation, thus efficient suppression is a prerequisite for successful transformation.

## 4. Experimental Section

### 4.1. Materials

Ten cultivars, which had above 80% shoot regeneration rates (Table S2), were used for *Agrobacterium*-mediated transformation experiments, including five US cultivars General, Kottman, Dennison, Williams 82 and Bert, and five Chinese cultivars Liaodou 10, Liaodou 14, Liaodou 16, Shennong 9 and Shennong 12.

### 4.2. Agrobacterium Preparation

For our experiment, the binary plasmid, pCAMBIA3301-1 with *GUS* and *BAR* genes was introduced into the super-virulent *Agrobacterium tumefaciens* strain EHA105, which provided by Agro-Biotechnology Research Institute of Jilin Academy of Agricultural Sciences, China. *A. tumefaciens* stocks of EHA105/pCAMBIA3301-1 was grown on solidified YEP medium (10 g·L^−1^ peptone, 5 g·L^−1^ yeast extract, 5 g·L^−1^ NaCl, 1.5% (*w*/*v*) agar (pH 7.0)) containing 25 mg·L^−1^ rifampicin and 50 mg·L^−1^ kanamycin and incubated at 28 °C until colony formation. Fifty milliliters liquid YEP medium containing antibiotics (same as above but without agar) was inoculated with a single colony and shaken at 28 °C, 180 rpm until the OD_600_ reached 0.8, −70 °C glycerol stocks. Before explant inoculation, 30 µL of *A. tumefaciens* glycerol stock was added to 5 mL liquid YEP medium containing antibiotics for 24 h at 28 °C, 180 rpm. Subsequently, 30 µL of the 5 mL starter culture was transferred to a 100 mL YEP culture, and grew overnight to OD_600_ = 0.8 at 28 °C, 180 rpm. The *A. tumefaciens* culture was centrifuged for 10 min at 3500 rpm and the pellet cell was resuspended in 1/10 Gamborg’s B5 medium with 3% (*w*/*v*) sucrose, 3.9 g·L^−1^ 4-Morpholineethanesulfonic acid (MES), filter-sterilized 0.25 mg·L^−1^ gibberellin A_3_ (GA_3_), 1.67 mg·L^−1^ 6-benzylaminopurine (6-BA), 400 mg·L^−1^ l-Cysteine (l-Cys), 154.2 mg·L^−1^ dl-Dithiothreitol (dl-DTT) and 200 µm As, pH 5.4.

### 4.3. Infection and Co-Cultivation of Explant

Soybean seeds were surface sterilized using chlorine gas made by mixing in 3.5 mL of 12 N HCl and 100 mL bleach (5.25% sodium hypochlorite) for 12 h (Figure S2A). Sterilized seeds were germinated on B5 medium with 2% (*w*/*v*) sucrose and 0.7% (*w*/*v*) agar, pH 5.8, under dark for 16 h, at 25 °C (Figure S2B). The seedling’s coat was removed, and then a longitudinal cut was made to separate two cotyledonary node explants. The primary shoot was subsequently removed and the cotyledonary node was wounded by cutting 6–8 times (Figure S2C). Explants were infected with *Agrobacterium* suspension in the shaker (126 rpm) for 30 min (Figure S2D). Thereafter, ten explants were cultured per 90 × 15mm petri dish, and the explants were placed on a filter paper laid over the co-cultivation medium, same as the resuspension medium described above, solidified with 0.7% (*w*/*v*) agar. Co-cultivation plates were incubated at 24 °C for 5 days in the dark (Figure S2E).

### 4.4. Transgenetic Shoots Induction and Plant Regeneration

After 5 days co-cultivation, the explants were briefly washed in liquid shoot induction medium containing Gamborg B5 salts, 3% (*w*/*v*) sucrose, 0.59 g·L^−1^ MES, pH 5.7 and filter-sterilized Gamborg B5 vitamins, 500 mg·L^−1^ carbenicillin, 1.67 mg·L^−1^ 6-BA were added after autoclaving. Explants were subsequently removed majority of the hypocotyl approximate 3–5 mm below the cotyledonary node and cultured on shoot induction medium (same as above but with 0.8% (*w*/*v*) agar and 5 mg·L^−1^ phosphinothricin) with the hypocotyl embedded in the medium and the cotyledonary node region facing upwards. The medium was changed every two weeks (Figure S2F). After four weeks, big shoots that may have developed from the primary shoot were cut and discarded. Explants were transferred to shoot elongation medium containing MS salts, 3% (*w*/*v*) sucrose, 0.3% (*w*/*v*) phytagel, 0.59 g·L^−1^ MES, pH 5.7 and filter-sterilized 50 mg·L^−1^ l-Asparagine (l-Asp), 50 mg·L^−1^ l-Glutamine (l-Glu), 0.5 mg·L^−1^ GA_3_, 0.1 mg·L^−1^ indole-3-acetic acid, 1 mg·L^−1^ zeatin, 250 mg·L^−1^ ticarcillin (Tic), 100 mg·L^−1^ cefotaxime (Cef), and 5 mg·L^−1^ phosphinothricin were added after autoclaving. The medium was changed every two weeks (Figure S2H). Culture conditions during shoot induction and elongation stages included an 18-h photoperiod at 28 °C. 2–3 cm long elongated shoots were placed into rooting medium comprised of MS salts, 2% (*w*/*v*) sucrose, 0.3% (*w*/*v*) phytagel, 0.59 g·L^−1^ MES, pH 5.6 and filter-sterilized 2 mg·L^−1^ indolebutyric acid, 50 mg·L^−1^ l-Asp, 50 mg·L^−1^ l-Glu, 250 mg·L^−1^ Tic, 100 mg·L^−1^ Cef were added after autoclaving (Figure S2I). Rooted seedlings were transferred to soil grown in the greenhouse.

### 4.5. Detection of T*_0_* Plants

Transgenic soybean plants were verified by LibertyLink strip analysis. LibertyLink strips (Envirologix, Portland, OR, USA) were used to determinate genetically modified plants containing the phosphinothricin *N*-acetyltransferase protein following the manufacturer’s instruction. Briefly, leaf tissue was ground and 0.25 mL of protein extraction buffer was added before regrinding. Development of the control line indicated that the strip had functioned properly, and the second line would show up when the tested sample was positive. As shown in Figure 1B, +: positive transgenic plants; −: negative transgenic plants.

### 4.6. β-Glucuronidase (GUS) Staining of Cotyledonary Node

After 5 days of co-cultivation, explants were first washed using sterile distilled water, then excised into cotyledonary node and immediately incubated with GUS histochemical stain (100 mM phosphate buffer (pH 7.0), 10 mM Na_2_EDTA (pH 8.0), 0.8% Triton-X, 0.5 mM K_4_Fe(CN)_6_, 0.5 mM K_3_Fe(CN)_6_, 1 mM X-gluc (Inalco, Milan, Italy)) overnight at 37 °C to verify T-DNA transfer. After staining, the explants were washed in 70% ethanol and assessed blue-staining areas.

### 4.7. BAR Gene Analysis during Co-Cultivation Period

The PCR analysis was conducted to determine *BAR* gene accumulation during co-cultivation period. Explants were washed 5 times using sterile distilled water and surface dried with a paper towel, then excised into cotyledonary node (approximately 5 mm above and below junction of the cotyledon and the hypocotyl) (Figure 1A), immediately frozen in liquid nitrogen and stored at −80 °C. Sampling times were before infection (0 HAI), 1 day after co-cultivation (1 DAC), 2 days after co-cultivation (2 DAC), 3 days after co-cultivation (3 DAC), 4 days after co-cultivation (4 DAC) and 5 days after co-cultivation (5 DAC), respectively. Genomic DNA was extracted using DNA Extracting Kit (Takara Bio Inc., Dalian, China). The 270-bp *BAR* gene coding region was amplified using a pair of primers: 5′-GCACCATCGTCAACCACTA-3′ and 5′-TCAGCAGGTGGGTGTAGAG-3′. The amplified products were isolated by electrophoresis on a 1% (*w*/*v*) agarose gel and photographed with the gel imaging system.

### 4.8. Measurement of Gibberellin (GA), Abscisic Acid (ABA), Zeatin Riboside (ZR) and Methyl Jasmonate (MeJA) Content

The extraction and purification of endogenous hormones used the method modified by Wang *et al.* [64]. Frozen samples (1 g) were ground in liquid nitrogen, then extracted and homogenised in 5 mL of cold 80% methanol (containing 40 mg·L^−1^ butylated hydroxytoluene as antioxidant) in darkness period at 4 °C overnight. The extract centrifuged at 12,000 rpm for 15 min at 4 °C. For purification, the supernatant was passed through C_18_ Sep-Pak cartridges (Waters Corp., Milford, MA, USA). The eluate was dried down by pure N_2_ at 35 °C, then dissolved in 2 mL of phosphate-buffered saline (PBS) (pH 7.5) containing 0.1% Tween-20 (*v*/*v*) and 0.1% (*w*/*v*) gelatin. Free GA, ABA, ZR and MeJA were quantified by Enzyme-linked Immunosorbent Assay (ELISA) Kit (College of Agronomy and Biotechnology, China Agricultural University, Beijing, China) following the protocol described by Yang *et al.* [65]. Calculations of ELISA data were performed as described by Weiler *et al.* [66].

### 4.9. Assay of Polyphenol Oxidase (PPO) and Peroxidase (POD) Activity

Extract of enzymes was prepared at 4 °C using the procedure with slight modifications as suggested by Sharma and Singh [67]. Frozen samples (0.5 g) were ground in liquid nitrogen, together with 6 mL of 50 mM phosphate buffer (pH 7.8) containing 10 mM Polyvinylpyrrolidone (PVP) were homogenized and centrifuged at 12,000 rpm for 30 min at 4 °C. An ammonium sulphate fraction was carried out. Firstly, ammonium sulphate was added into extracted supernatant to give 30% saturation and centrifuged at 12,000 rpm for 30 min at 4 °C to remove PVP. The supernatant was removed into a new tube, secondly the ammonium sulphate fraction precipitating between 45% and 95% saturation was collected and redissolved. Total protein concentration in soluble enzyme extracts was determined using the Bradford [68] assay. Bovine serum albumin (BSA) was used as a standard.

PPO activity was assayed by the modification of the technique described by Concellón *et al.* [69]. The assay medium contained 1 mL enzyme extract and 3 mL of 100 mM catechol. The altering in absorbance at 410 nm was monitored at 30 s intervals for 3 min using spectrophotometer, and the average change in absorbance per min, were calculated. PPO activity was expressed as ΔA_410_·min^−1^·mg^−1^ protein.

POD was measured according to the method of Reuveni [70]. The reaction mixture (1.2 mL) contained 0.1 M phosphate buffer (pH 7.0), 4 mM guaiacol and 0.4 mL enzyme extract. The reaction was initiated by adding 3 mM H_2_O_2_. The increase in absorbance at 470 nm was measured using spectrophotometer. Levels of enzyme activity were expressed as ΔA_470_·min^−1^·mg^−1^ protein.

### 4.10. Expression of Selected Genes

There are three groups of selected genes used in this study. The first group of genes (*GA20ox2*, *CYP707A2*, *IPT5*, *OPR3*) encode key enzymes involved in hormone metabolism [40,41,42,43,46]. The second group of genes (*PPO1*, *PRX71*) encode PPO and POD, respectively [47,48]. The third group of genes (*CYCD3*, *CYCA3*) work in relation to cell cycle regulation [44,45]. These genes have been studied in *Arabidopsis* or *Medicago*, but have not been investigated in soybean. We obtained soybean genes that were homologous with *Arabidopsis* or *Medicago*. The information of selected genes can be found in Table S3. RNA was isolated from sampled cotyledonary nodes utilizing Plant Total RNA Isolation Kit (Qiagen, Hilden, Germany). Synthesis of cDNA was performed with a SuperScript III first-strand synthesis system (Invitrogen, Carlsbad, NM, USA) using 2 μg of RNA, and 1 μL of the reaction mixture was subsequently used for real-time quantitative PCR in a 50 μL reaction volume using SYBR Green I (Takara Bio Inc.). Primers in Table S4 were used to amplify specific genes. *EF1α* gene was used as a calibrator. The following thermal cycle conditions were used: 95 °C for 30 s, followed by 35 cycles of 95 °C for 5 s, 58 °C for 20 s, and 72 °C for 20 s. The qRT-PCR experiments were performed with three biological replications, and each biological replication was measured in three technical replications. Following the PCR, a melting curve analysis was performed. Threshold cycle was used for relative quantification of the input target number. Relative fold difference (N) is the number of infected target gene transcript copies relative to the uninfected control gene transcript copies and is calculated as follows:

*N* = 2^ΔΔ*C*t^ = 2^(Δ*C*t *infected* − Δ*C*t *control*)^ (1)

where ΔΔ*C*_t_ = Δ*C*_t_ of the infected (1 DAC, 3 DAC and 5 DAC) samples minus Δ*C*_t_ of the uninfected control (0 HAI) samples, and Δ*C*_t_ is the difference in threshold cycles for the target gene and the *EF1α* internal reference.

### 4.11. Measurement of DNA Quantity

4′-6-Diamidino-2-phenylindole (DAPI) is utilized extensively in detecting and quantifying of DNA. Fluorescence of DAPI-stained DNA is proportional to DNA quantity. In this experiment, the cotyledonary node samples were fixed in FAA (50% ethanol, 5% glacial acetic acid, 10% formalin) before embedded in Steedman’s wax [71], and then cut into ribbon sections. Samples were stained for 15 min with 1 µg·mL^−1^ DAPI in McIlvaine’s buffer, then washed three times for 15 min each with Mcllvaine’s buffer. Fluorescence microscope was used to observe the meristem of cotyledonary node, and took pictures. Fluorescent pictures were analyzed using Image J software (The National Institutes of Health, Madison, WI, USA), fluorescence of individual nuclei was measured to carry out DNA semiquantitative analysis. Average optical density was used as a measurement of DNA content standards, which is calculated as follows:

AOD (average optical density) = IOD (integrated optical density)/Area
(2)

### 4.12. Statistical Analysis

The assays of endogenous hormones, enzymes and genes were determined in three biological replications and each biological replication was measured in triplicate, the means and standard deviations were calculated. The significance analysis was performed by Duncan’s new multiple-range test in DPS v7.05 statistical software (Hangzhou Reifeng Information Technology Co., Ltd., Hangzhou, China).

## 5. Conclusions

Our results indicate that soybean genotype-dependency is mainly caused by genotypes’ susceptibility to *Agrobacterium*. There is a tangible and complex link between the plant factors that participate in the response mechanism of *Agrobacterium*-plant interaction. We suggest that in cotyledonary nodes GA and ZR are positive plant factors for *Agrobacterium*-mediated soybean transformation by facilitating germination and growth and increasing the number of cells that in DNA synthesis cycle, respectively; whereas ABA, MeJA, PPO and POD are negative plant factors for *Agrobacterium*-mediated soybean transformation by repressing germination and growth, and inducing defence reactions, respectively.

## Supplementary Materials

Supplementary materials can be found at http://www.mdpi.com/1422-0067/16/08/18522/s1.
