# Supplementary material for: Comparison of Soybean Transformation Efficiency and Plant Factors Affecting Transformation during the Agrobacterium Infection Process
Source: Int J Mol Sci. 2015 Aug 7;16(8):18522–43. doi: 10.3390/ijms160818522 (PMC4581258; doi:10.3390/ijms160818522)
Supplement: Supplementary file 1 [file ijms-16-18522-s001.pdf]

Supplementary Information

Table S1. Endogenous hormone content and enzyme activity in cotyledonary nodes from different genotypes.

Endogenous Hormone, Enzyme	Genotype	Endogenous Hormone Content (ng·mg^{-1} FW) and Enzyme Activity (△A410·min^{-1}·mg^{-1} Protein, △A470·min^{-1}·mg^{-1} Protein) in Different Phases			
		0 HAI	1 DAC	3 DAC	5 DAC
GA	General	7.13 ± 0.21 ^{c}	7.46 ± 0.29 ^{e}	5.53 ± 0.03 ^{e}	5.79 ± 0.87 ^{a}
	Liaodou 16	8.51 ± 0.69 ^{ab}	8.55 ± 0.83 ^{cd}	6.29 ± 0.08 ^{cd}	3.18 ± 0.41 ^{d}
	Kottman	8.26 ± 0.28 ^{ab}	8.07 ± 0.06 ^{de}	7.33 ± 0.42 ^{b}	4.18 ± 0.33 ^{c}
	Williams 82	7.73 ± 0.34 ^{bc}	9.08 ± 0.48 ^{bc}	6.51 ± 0.27 ^{c}	4.62 ± 0.3 ^{bc}
	Shennnong 9	8.06 ± 0.55 ^{ab}	9.89 ± 0.60 ^{ab}	5.93 ± 0.05 ^{de}	5.53 ± 0.32 ^{ab}
	Bert	8.95 ± 0.36 ^{a}	10.18 ± 0.52 ^{a}	8.48 ± 0.32 ^{a}	5.09 ± 0.5 ^{abc}
ABA	General	664.92 ± 57.37 ^{c}	730.27 ± 42.48 ^{b}	716.50 ± 28.07 ^{a}	293.08 ± 9.99 ^{d}
	Liaodou 16	951.90 ± 61.98 ^{b}	850.90 ± 7.19 ^{a}	499.53 ± 7.46 ^{b}	385.45 ± 25.28 ^{b}
	Kottman	940.73 ± 65.11 ^{b}	865.79 ± 52.46 ^{a}	452.73 ± 20.82 ^{c}	490.57 ± 12.07 ^{a}
	Williams 82	645.31 ± 54.88 ^{c}	448.55 ± 62.23 ^{d}	404.14 ± 15.66 ^{d}	333.07 ± 4.84 ^{c}
	Shennnong 9	1028.49 ± 21.37 ^{ab}	617.37 ± 15.97 ^{c}	337.48 ± 34.29 ^{d}	288.10 ± 4.72 ^{d}
	Bert	1118.39 ± 27.39 ^{a}	668.54 ± 63.44 ^{bc}	448.61 ± 26.54 ^{c}	404.17 ± 14.76 ^{b}
MeJA	General	120.08 ± 9.95 ^{ab}	303.99 ± 5.49 ^{a}	154.83 ± 1.48 ^{b}	113.70 ± 14.41 ^{bc}
	Liaodou 16	90.76 ± 2.03 ^{c}	252.61 ± 9.35 ^{b}	171.40 ± 12.12 ^{a}	133.33 ± 2.32 ^{a}
	Kottman	126.17 ± 11.18 ^{a}	316.62 ± 13.24 ^{a}	151.79 ± 7.88 ^{b}	99.50 ± 6.08 ^{c}
	Williams 82	87.74 ± 2.76 ^{c}	132.10 ± 2.93 ^{d}	106.20 ± 7.88 ^{c}	101.58 ± 12.4 ^{c}
	Shennnong 9	111.69 ± 6.61 ^{b}	169.82 ± 9.28 ^{c}	143.72 ± 5.25 ^{b}	125.62 ± 2.36 ^{ab}
	Bert	63.98 ± 1.76 ^{c}	103.59 ± 5.22 ^{e}	85.41 ± 8.38 ^{d}	53.44 ± 9.87 ^{d}
ZR	General	16.31 ± 1.24 ^{bc}	20.94 ± 1.24 ^{b}	20.84 ± 0.79 ^{b}	16.80 ± 1.47 ^{d}
	Liaodou 16	20.70 ± 0.61 ^{a}	10.31 ± 0.64 ^{d}	16.31 ± 0.99 ^{c}	15.06 ± 0.78 ^{d}
	Kottman	21.76 ± 1.60 ^{a}	16.53 ± 0.64 ^{c}	19.46 ± 1.23 ^{b}	15.79 ± 2.15 ^{d}
	Williams 82	15.11 ± 1.58 ^{cd}	27.12 ± 1.10 ^{a}	27.09 ± 0.93 ^{a}	23.02 ± 1.69 ^{b}
	Shennnong 9	18.17 ± 1.26 ^{b}	27.59 ± 1.07 ^{a}	28.46 ± 1.79 ^{a}	26.32 ± 0.71 ^{a}
	Bert	13.64 ± 1.51 ^{d}	26.26 ± 1.26 ^{a}	26.49 ± 1.82 ^{a}	19.78 ± 0.74 ^{c}
PPO	General	0.017 ± 0.001 ^{b}	0.103 ± 0.001 ^{ab}	0.124 ± 0.003 ^{a}	0.125 ± 0.001 ^{a}
	Liaodou 16	0.025 ± 0.003 ^{a}	0.112 ± 0.006 ^{a}	0.117 ± 0.006 ^{b}	0.110 ± 0.004 ^{b}
	Kottman	0.014 ± 0.001 ^{b}	0.101 ± 0.007 ^{b}	0.120 ± 0.001 ^{ab}	0.114 ± 0.003 ^{b}
	Williams 82	0.016 ± 0 ^{b}	0.068 ± 0.006 ^{c}	0.093 ± 0.001 ^{c}	0.069 ± 0.005 ^{d}
	Shennnong 9	0.016 ± 0.001 ^{b}	0.057 ± 0.006 ^{d}	0.084 ± 0.004 ^{d}	0.087 ± 0.001 ^{c}
	Bert	0.017 ± 0.002 ^{b}	0.073 ± 0.006 ^{c}	0.097 ± 0.001 ^{c}	0.091 ± 0.001 ^{c}
POD	General	0.075 ± 0.007 ^{c}	1.378 ± 0.04 ^{a}	2.819 ± 0.086 ^{b}	3.530 ± 0.103 ^{b}
	Liaodou 16	0.012 ± 0.001 ^{e}	1.310 ± 0.069 ^{a}	3.286 ± 0.126 ^{a}	4.249 ± 0.191 ^{a}
	Kottman	0.082 ± 0.005 ^{c}	0.732 ± 0.061 ^{d}	2.666 ± 0.074 ^{c}	3.184 ± 0.134 ^{c}
	Williams 82	0.025 ± 0.002 ^{d}	0.518 ± 0.066 ^{e}	1.684 ± 0.079 ^{e}	2.689 ± 0.042 ^{d}
	Shennnong 9	0.111 ± 0.005 ^{b}	1.158 ± 0.028 ^{b}	2.026 ± 0.047 ^{d}	2.105 ± 0.062 ^{e}
	Bert	0.181 ± 0.009 ^{a}	0.87 ± 0.036 ^{c}	2.169 ± 0.051 ^{d}	2.285 ± 0.087 ^{e}

The data represent means ± SD based on three biological replications. Values within different letters are significant difference at 0.05 levels for different genotypes at the same time.

Table S2. Shoot regeneration rates of 27 cultivars.

Cultivar	No. of Explants	No. of Explants with Multiple Shoots	Shoot Regeneration Rate (%)
Shennong 9	100	96	96.00
Bert	98	90	91.84
Liaodou 14	100	91	91.00
Kottman	100	93	93.00
Williams 82	101	89	88.12
Liaodou 16	100	85	85.00
Dennison	102	85	83.33
General	101	83	82.18
Shennong 12	99	85	85.86
Liaodou 10	101	89	87.82
Shennong 20-41	101	78	77.23
Dilworth	101	80	79.21
Tiefeng 31	102	76	74.51
Darby	105	82	78.10
Shennong 3	102	78	76.47
Amsoy	103	79	76.70
Shennong G23-4	101	76	75.25
Liaodou 3	102	77	75.49
Tiffin	99	74	74.75
Tiefeng 33	105	66	62.86
Shennong 20-10	101	61	60.40
Liaodou 12	102	54	52.94
HS93-4118	101	53	52.48
Shennong 20-34-2	103	50	48.54
Wyandot	100	50	50.00
Shennong 96-3-1-1-1	102	25	24.51
Yongwei 6	101	25	24.75

Table S3. Soybean gene locus and *Arabidopsis* or *Medicago* homologues.

Gene Name	Soybean Locus [1]	*Arabidopsis* or *Medicago* Locus [1]	Gene Name	Amino Acid Identity
GmGA20OX2	Glyma03g02260	AT5G51810	*GA20OX2*	72.2%
GmCYP707A2	Glyma17g36070	AT2G29090	*CYP707A2*	69.5%
GmIPT5	Glyma17g02080	AT5G19040	*IPT5*	64.2%
GmOPR3	Glyma11g00980	AT2G06050	*OPR3*	68.3%
GmPPO1	Glyma15g07710	Medtr2g013030	*PPO1*	74.7%
GmPRX71	Glyma08g19170	AT5G64120	*PRX71*	71.0%
GmCYCD3	Glyma05g20990	AT5G67260	*CYCD3*	66.8%
GmCYCA3	Glyma14g09610	AT5G43080	*CYCA3*	72.1%

[1] Obtained from the phytozome v9.1 database (http://phytozome.jgi.doe.gov/pz/portal.html).

Table S4. qRT-PCR primer sequences.

Gene Name	Forward (5′–3′)	Reverse (5′–3′)
GmGA20OX2	AACACTTGATGCTTTCTCGAGGTG	TGGCCATGGAGCAATTGTGTG
GmCYP707A2	GTCACCAAGTTTCGGTGGGAAGTG	TGGGAGTCCATTCAGTGGCAAG
GmIPT5	TCACCACCGCAACAACAAGGAG	AATTGCGTGGCGAGGTCTATTGC
GmOPR3	AACCGCTCAAGGGTATCCACAC	GTTTCCACGCTTGGACTTGCTG
GmPPO1	TCTATCCTTCGTGCCCACAGTC	AAATGGATGCAACGGAGAAGGG
GmPRX71	AGACCTCGTCATTCTTGCTGGTG	TGTCTGCGAAAGATCGGCAAGC
GmCYCD3	AGCCGCAGTTGAAGAGGAGAAG	TTGACACACGACTAACAGAAGGC
GmCYCA3	AAAGACACGACCCGCAACTC	CTTACTAGGATCCACCTCCATCCC

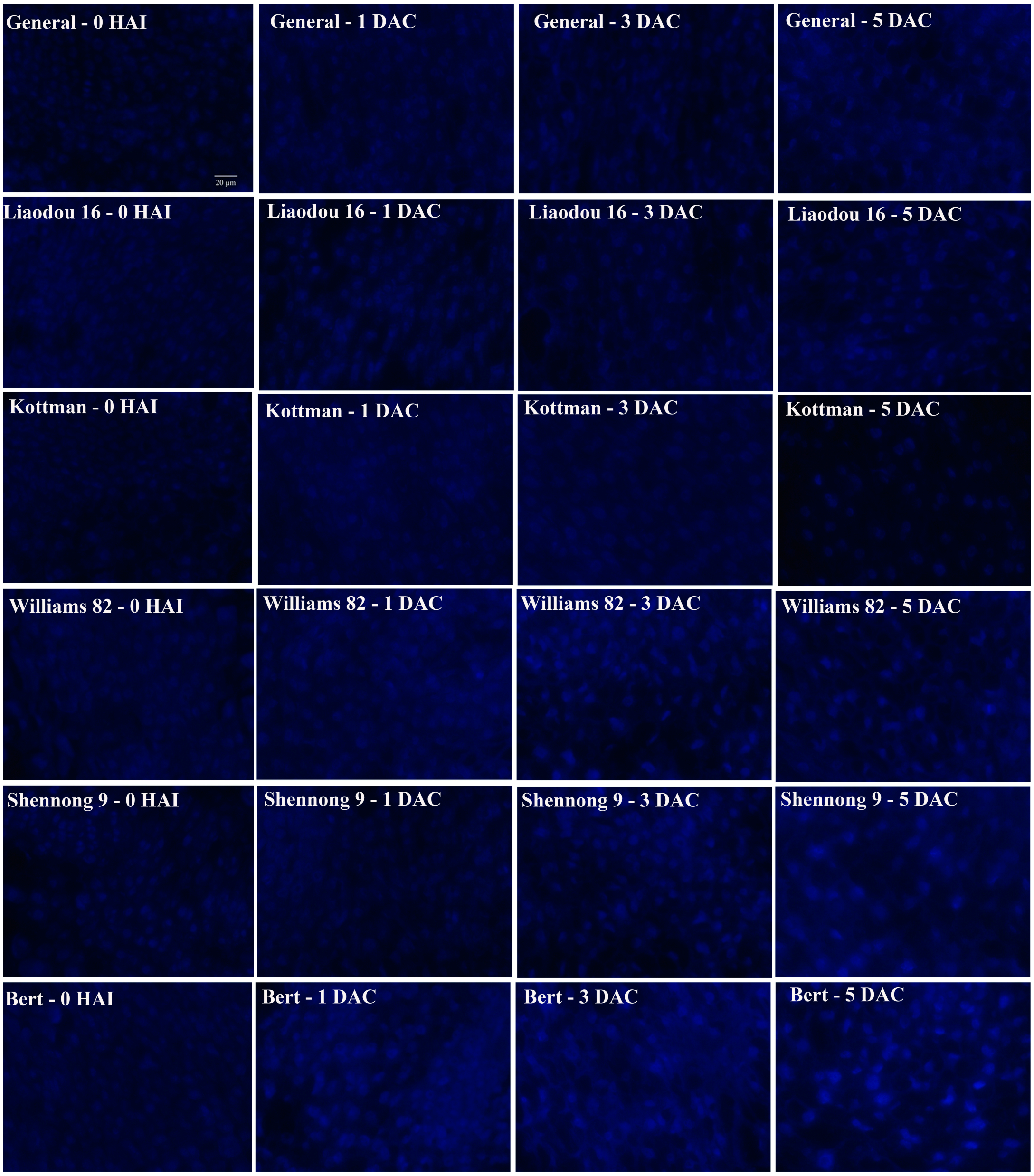

Figure S1. Fluorescence of DAPI-stained nuclei in meristem of cotyledonary node from different genotypes during co-cultivation period, 0 HAI (0 h after infection), 1 DAC (1 day after co-cultivation), 3 DAC (3 days after co-cultivation) and 5 DAC (5 days after co-cultivation), Scale Bar = 20 μm.

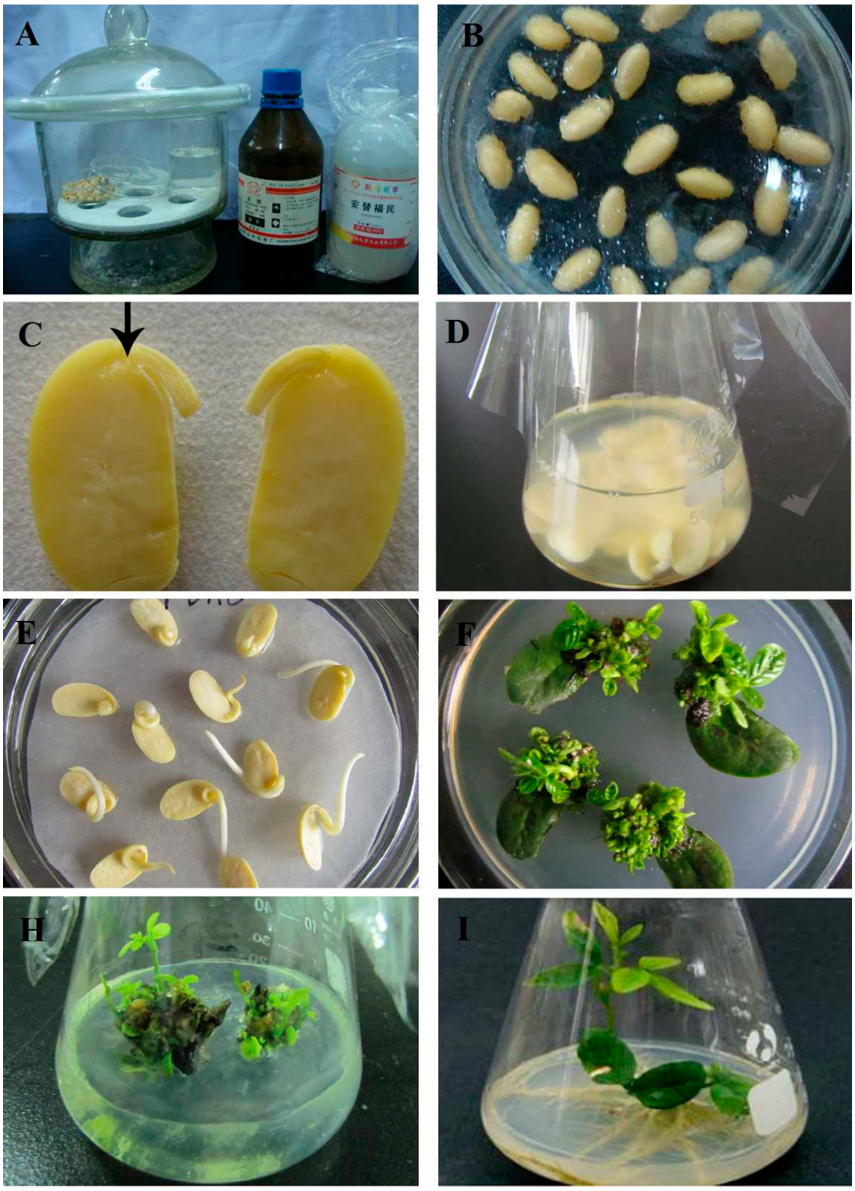

Figure S2. *Agrobacterium*-mediated transformation of cotyledonary node explants and regeneration, (**A**) Seed sterilization; (**B**) Seed germination; (**C**) Preparation of cotyledonary node explants, arrow means the site for wounding; (**D**) Infection of explants; (**E**) Co-cultivation of explants; (**F**) Transgenetic shoots selection; (**H**) Transgenetic shoots elongation; and (**I**) Rooted seedlings.
